# Gap and Inequality in the Economic Income of Independent Workers in the Region of Puno-Peru and the Effect of the Pandemic, 2019–2020

**DOI:** 10.3389/fsoc.2022.858331

**Published:** 2022-04-13

**Authors:** Julio C. Quispe Mamani, Giovana A. Flores Turpo, Dominga A. Calcina Álvarez, Cristóbal R. Yapuchura Saico, Wily L. Velásquez Velásquez, Santotomas L. Aguilar Pinto, Betsy Quispe Quispe, Nelly B. Quispe Maquera, Balbina E. Cutipa Quilca

**Affiliations:** ^1^Facultad de Ingeniería Económica, Universidad Nacional del Altiplano, Puno, Peru; ^2^Universidad Nacional Intercultural Fabiola Salazar Leguia de Bagua, Bagua, Peru; ^3^Facultad de Educación, Universidad Nacional Amazónica de Madre de Dios, Puerto Maldonado, Peru; ^4^Universidad Nacional Tecnológica de Lima Sur, Lima, Peru; ^5^Facultad de Ciencias Administrativas, Universidad Andina Néstor Cáceres Velásquez, Juliaca, Peru; ^6^Facultad de Ciencias de la Salud, Escuela Profesional de Odontología, Universidad Nacional del Altiplano, Puno, Peru; ^7^Facultad de Ciencias Contables, Escuela Profesional de Ciencias Contables, Universidad Nacional del Altiplano, Puno, Peru

**Keywords:** poverty, pandemic (COVID-19), education, living conditions, labor market

## Abstract

**Objective:**

This article seeks to determine the social determinants of inequality in economic income in independent workers in the Puno region in the periods 2019 and 2020.

**Methods:**

For which the quantitative approach was used, with descriptive and correlational design, considering the multiple regression model.

**Results:**

It was determined that there is a very significant income gap by educational level due to the productive differential that coronavirus disease 2019 (COVID-19) affected all the households; there is inequality in the economic income of independent workers, since in 2019, there was a greater inequality of economic income among independent workers (Gini = 0.6142) in relation to the national level (Gini = 0.415) and in 2020, the inequality of economic income increased due to COVID-19 problem, where the Gini coefficient amounted to 0.7136 in relation to the national level (Gini = 0.431).

**Conclusion:**

The determining factors of the economic income of the independent worker in the region of Puno in the periods 2019 and 2020 are the age that explains in 5.19 and 1.72%, the level of education that explains in 20.74 and 34.86% and the sex that explains in 37 and 14.19%, respectively.

## Introduction

The current scenario of the economic crisis that the world has been facing due to the pandemic of coronavirus disease 2019 (COVID-19), generated by severe acute respiratory syndrome coronavirus 2 (SARS-CoV-2), has shown the true reality of the economic and social conditions of households in the world (CEPAL, 2020; Kim and Kim, 2021; Zheng and Walsham, 2021); in developing countries, it worsened with greater complexity, given that poverty increased in the different social strata, showing a fragility in the indicators of this, where the most affected were the areas of education, economy, and health, since when entering the scenario of social confinement, they directly affected the economic and social development of their regions and their local territory (Witteveen, 2020; Aspachs et al., 2021); evidencing the vulnerability and inequality in economic income, access to public and social services, among others, directly affecting monetary and non-monetary poverty, weakening the social cohesion of its population, mainly in the marginal rural and urban areas (Marmot et al., 1997; Charles Coll et al., 2018; Beaunoyer et al., 2020; Agarwal, 2021; Bertogg and Koos, 2021; Boschken, 2021; Nguyen et al., 2021; Paz-Maldonado et al., 2021).

The indicators of the social and economic aspect in the world in 2020 show that 8.8% of total working hours were lost on average, equivalent to 255 million full-time workers, showing a decrease in 144 million jobs compared to 2019, evidencing that around 108 million workers are currently in the situation of extreme or moderately poor, where heads of household and their members are surviving on less than $3.20/day in purchasing power parity terms (Martín-Moreno et al., 2020; Alfani, 2021; Sun et al., 2021).

The existence of an increase in unemployment due to social confinement in times of COVID-19 caused many workers and mainly the independent to be inactive until today and despite seeking to insert themselves into the labor market, they are often not finding a secure and permanent job (Martín-Moreno et al., 2020; Aspachs et al., 2021).

In 2020, the number of unemployed reached 11.6 million people with an increase in unemployment of 3.4%, generating a loss in the economic income of households and the increase in poverty by at least 4.4% compared to 2019, gross domestic product (GDP) decreased by 5.3%; it becoming a period with a high rate of vulnerability and exposure to the reduction of their economic income due to COVID-19, the same that on average reached 27% of workers (Alvarez and Harris, 2020; Álvarez Marinelli et al., 2020; Peñafiel-Chang et al., 2020; del Río Lozano and del García Calvente, 2021).

In Latin America and the Caribbean, this behavior was similar, where 10% of rich individuals earned 22 times more than 10% of the group of poor individuals and only 1% who are in the situation of being rich own more than 20% of the national income; evidencing in this way that the female gender group earned less than the male group and the Afro-descendants and indigenous people earned less than the others, generating a clear inequality and inequality in the economic and social conditions between Latin Americans and Caribbeans (CEPAL, 2019; Álvarez Marinelli et al., 2020; Filgueira et al., 2020; Lugo et al., 2020). The aforementioned is the result of the type of education and the sector where they receive such training (private or public), providing greater opportunities and possibilities to those with better economic conditions, thus guaranteeing greater access to quality employment in the formal labor market and this is perpetual in some social groups (Charles Coll et al., 2018; Filgueira et al., 2020; Weller et al., 2020; Chiodi, 2021; Hossain, 2021).

Studying the behavior of the independent worker under these conditions is important, since at the level of Latin America by 2020 almost 65% of independent professionals are part of the age group between 21 and 40 years, where 50.3% of the total are male and 49.7% are female; of this, 57% work full time and 43% work part time, specifically affecting the latter group more categorically. Therefore, the impact of the pandemic was not the same between countries and this depends on the heterogeneities that each country has in the labor force and its characteristics that these have such as working conditions, the type of employment contract, and the characteristics of companies, small and microenterprises (Ahmed et al., 2020; Bidegain et al., 2020; Filgueira et al., 2020; Lugo et al., 2020; Peñafiel-Chang et al., 2020; Sierra, 2020; Weller et al., 2020; Chiodi, 2021).

This situation in Peru is more critical, given that by 2020 almost 32,330,000 people could not guarantee to cover their basic needs, since poverty increased by 10% compared to 2019, turning the vulnerable population group into poor to precariousness (Cornejo Urbina, 2020; Lugo et al., 2020; Mújica and Pachas, 2021). The region of Lima was the most affected at the country level, in view of the fact that the number of people who cannot cover the expenses for the food basket increased; since in 2019, 14% of the population was registered that was in the situation of poverty and increased to 27.5% in 2020, affecting 45.7% of the rural population, since in that area it increased by 4.9% compared to 2019 and by 26% of the urban population, due to the increase in 11% (Oblitas Gonzales and Sempertegui Sánchez, 2020; Villar-mayuntupa and Villar-mayuntupa, 2020; Chiodi, 2021; Córdoba Ruíz and Lin, 2021; Gómez-Arteta, 2021).

Salaried workers make up 46.3% of the total and self-employed workers are part of 37%; so, those most affected by COVID-19 are in the urban area and are part of the salaried, since they represent more than half of the workers, followed by the independent who reach 34.2%. In this sense, despite the fact that in 2019, 21.0% of independents reached a level of university and non-university higher education and 47.8% of salaried workers in 2019 reached on average an income level of S/. 895.00 soles, reaching half of the dependents that was S/. 1,804.00 soles; but thanks to the restrictions that were implemented by COVID-19, this allowed to lose almost half of the job, directly affecting the economic income of these households and their members (Bidegain et al., 2020; Jaramillo and Ñopo, 2020; Jaramillo et al., 2020; Santa María et al., 2020; Villar-mayuntupa and Villar-mayuntupa, 2020; Huaman, 2021; Mújica and Pachas, 2021).

It can be determined that the situation of inequality in the region of Puno and Peru increased in the last two decades and strongly in 2020, since the existence of monetary inequality is remarkable, while poverty and inequality are multidimensional, which include the health sector, education, housing, life expectancy; thanks to the pandemic, which is aggravated, since poverty increases abruptly and can contribute toward the regression of the regions and the country takes a decade in overcoming poverty and the search for equity and equality in income, if it does not take measures to avoid it (Quispe Mamani et al., 2020; Apaza-Panca et al., 2021; Mejia, 2021).

In this sense, the questions that the present research sought to answer were: What are the social determinants of inequality in economic income in independent workers in the Puno region in the periods 2019 and 2020?, Is there a gap in economic income by gender and level of education in independent workers in the Puno region in the periods 2019 and 2020?, and Is there inequality in the economic income of independent workers in the Puno region in the periods 2019 and 2020?

The objective of this research was to determine the social determinants of inequality in economic income in independent workers in the Puno region in the periods 2019 and 2020, in addition to determining the gap in economic income by gender and level of education in independent workers in the Puno region in the periods 2019 and 2020, and determine the inequality in the economic income of independent workers in the Puno region in the periods 2019 and 2020.

The hypotheses to be tested were that the social determinants of the inequality in economic income in the independent workers in the region of Puno in the periods 2019 and 2020 are the age of the head of household, level of education achieved by the worker, and the gender of the worker; in addition, there is a gap in economic income by gender and level of education among independent workers in the Puno region in the periods 2019 and 2020 and there is inequality in the economic income of independent workers in the Puno region in the periods 2019 and 2020.

## Materials and Methods

### Type and Design of Research

The approach to which the research corresponds is quantitative, with descriptive and correlational research design; in addition, it corresponds to the type of non-experimental research (Sampieri and Collado, 2010).

### Población

Considering the database of the National Household Survey (ENAHO), the module “employment and income” and “education,” the population under study corresponds to all the independent workers of the Puno region that includes the 13 provinces and the 110 districts, classified by type of main occupation, residents in the urban and rural areas. In addition, it did not consider members of the armed forces who live in barracks and camps nor people who reside in collective housing, such as hotels, hospitals, asylums and religious cloisters, prisons, among others.

### Sample Type and Data Source

According to the National Institute of Statistics and Informatics (INEI), the sample considered by the database of the National Household Survey (ENAHO) of 2019 and 2020 is of the type of sampling is probabilistic, of areas, stratified, multistage, and independent in each province of study at the level of the region of Puno, considering a level of confidence of the sample results of 95%. This study sample amounts to 213 heads of household. It should be noted that for the determination of the sample, the methodology applied by the INEI was considered, the same as that validated by that institution.

### Econometric Model

The ordinary least squares regression (MCO) model was considered but according to the traditional Mincer equation, where a semilogarithmic model is estimated, using as a dependent variable, the logarithm of the economic income of the independent workers and as independent variables such as the level of education, the sex of the respondent, the age of the worker, and the square of this, in the area of the Puno region in the periods 2019 and 2020; it is detailed below:


$$\begin{array}{l} \text{Ln}(\text{Income of the }\\ \text{self}-\text{employed}){\text{= β}}_{\text{0}}{\text{+β}}_{\text{1}}\text{Level of education of the self }\\ \;-{\text{employed+β}}_{\text{2}}\text{Age of the self}-\text{employed }\\ {\text{ +β}}_{\text{3}}\text{Age of the self}-{\text{employed}}^{2}\;\\ {\text{ +β}}_{\text{4}}\text{Sex of the self}-\text{employed worker+μ} \end{array}$$

The details of the characteristics of the variables are shown in Table 1.

**Table 1:** Identification of analysis variables.

**Descripción**	**Descripción de las variables**	**Fuente de dato**
Income of the self-employed	Natural logarithm of monthly income	INEI, National Household Survey (ENAHO) Database
Level of education of the self-employed	1 = Incomplete Primary, 2 = Complete Primary, 3 = Incomplete Secondary, 4 = Complete Secondary, 5 = Incomplete Non-University Superior, 6 = Complete Non-University Superior, 7 = Incomplete University Superior, 8 = Complete University Superior, 9 = Postgraduate	
Years of education received	Years of education achieved by the worker, the more years of education the greater return on income	
Age of the self-employed	Age in years completed	
Sex of the self-employed worker	1 = if male, 0 = if female	

*Source: Own elaboration with information from ENAHO-INEI-2019 and 2020*.

### Variable Analysis

The variables considered are the income of the independent worker by main occupation, sex, and age of the independent worker and level of education of the independent worker; the same that is given in Table 1, where the notation and description of the variables under analysis are identified.

## Results

According to the economic theory of the labor market, independent workers are those people who develop economic activities on their own, using their own capital and economic resources, in addition to these are generally people who know a trade and their income depends on the level of sales or provision of services that they perform in a certain period of time. In this sense, question 530 of the module “employment and income” of the ENAHO base refers to the monthly net income in soles by main occupation of the self-employed.

### Analysis of the Gap in Economic Income by Gender and Level of Education in Independent Workers in the Puno Region in the Periods 2019 and 2020

**Figure 1:**
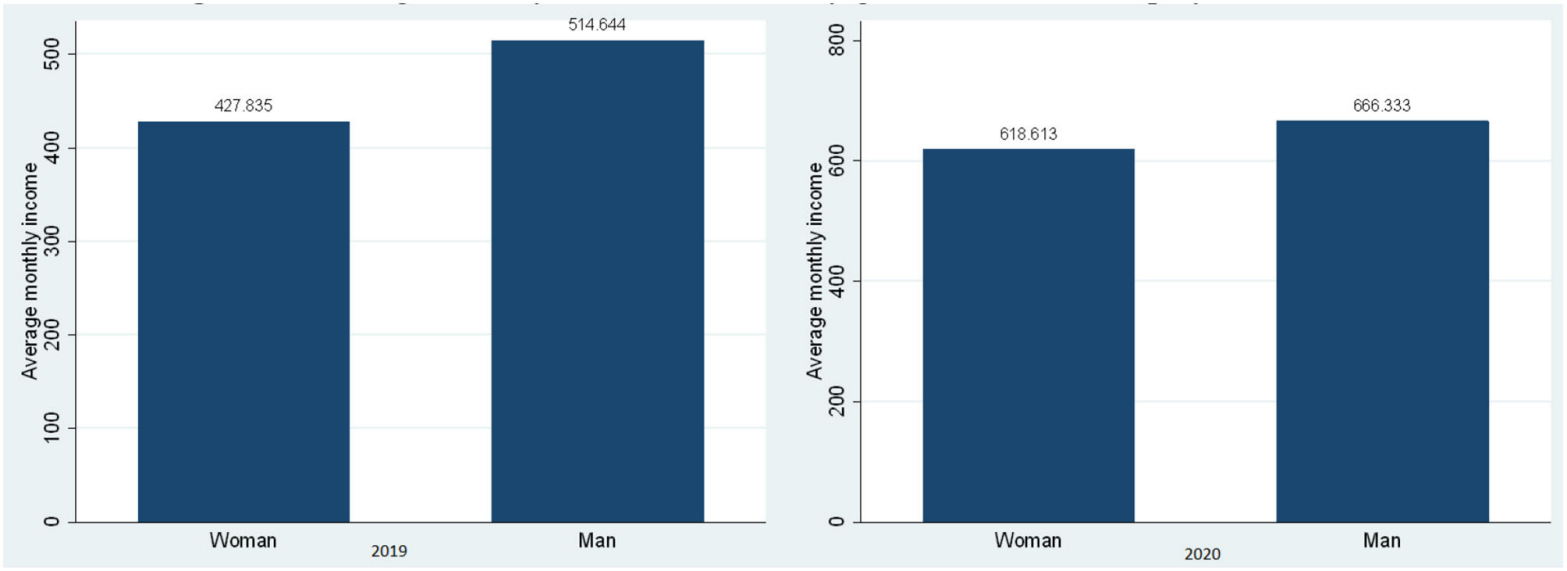
Average monthly economic income by gender of the self-employed, 2019–2020. Source: Own elaboration with information from ENAHO-INEI-2019 and 2020.

In Figure 1, the existence of the gap in the average monthly income by gender of dependent workers in the years 2019 and 2020 can be verified, where when applying the test of averages for the year 2019 in the scenario without pandemic, a substantial difference in the economic income of S/ is shown. 86.81 soles the same that is probably due to observable factors such as the level of education, work experience acquired between both the genders and to non-observable factors such as gender wage discrimination; on the contrary, by 2020, in times of pandemic, this gap reached the amount of S/. 47.72 soles, showing a considerable decrease, since the implementation of confinement and social restriction policies throughout the country restricted access to the provision of skilled and unskilled labor at the level of the different labor markets, which allowed to show a decrease in access to job opportunities.

When comparing the average monthly income in the scenario without pandemic and with pandemic by years and between the same genders, a substantial improvement can be clearly seen, but the highlight in this case is that women showed a more considerable improvement compared to men, where it was S/. 190.78 soles and men only improved the amount of S/. 151.69 soles; this is justified because the government has so far benefited from social assistance programs through subsidiary bonds, where most of them have access to women and the population with limited economic resources that are part of the population group in a situation of poor and extreme poor.

**Figure 2:**
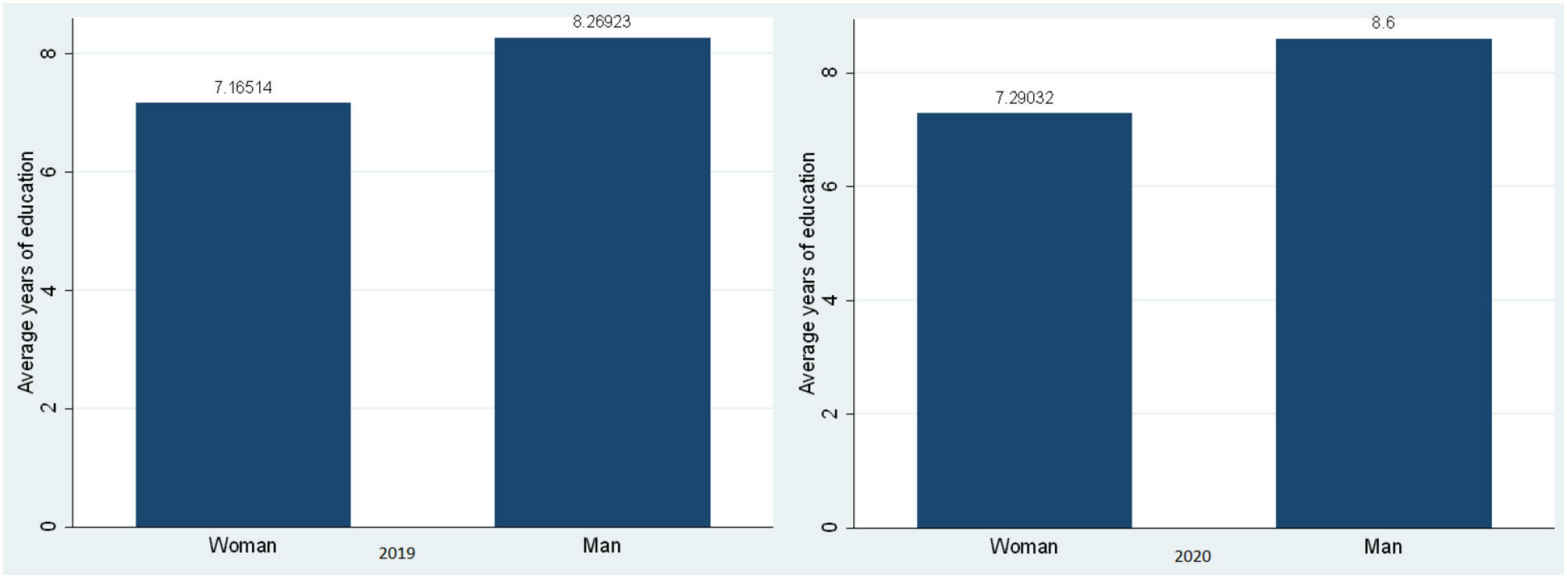
Average years of education of self-employed workers by gender, 2019–2020. Source: Own elaboration with information from ENAHO-INEI-2019 and 2020.

Figure 2 shows the gap in the educational level achieved, where you can observe slight differences in the average level of schooling achieved by men and women in both the 2019 and 2020. In fact, men managed to study on average 8 years in 2019 and this increased to 9 years in 2020, while women in 2019 reached 7 years of study and in 2020 this was maintained.

**Figure 3:**
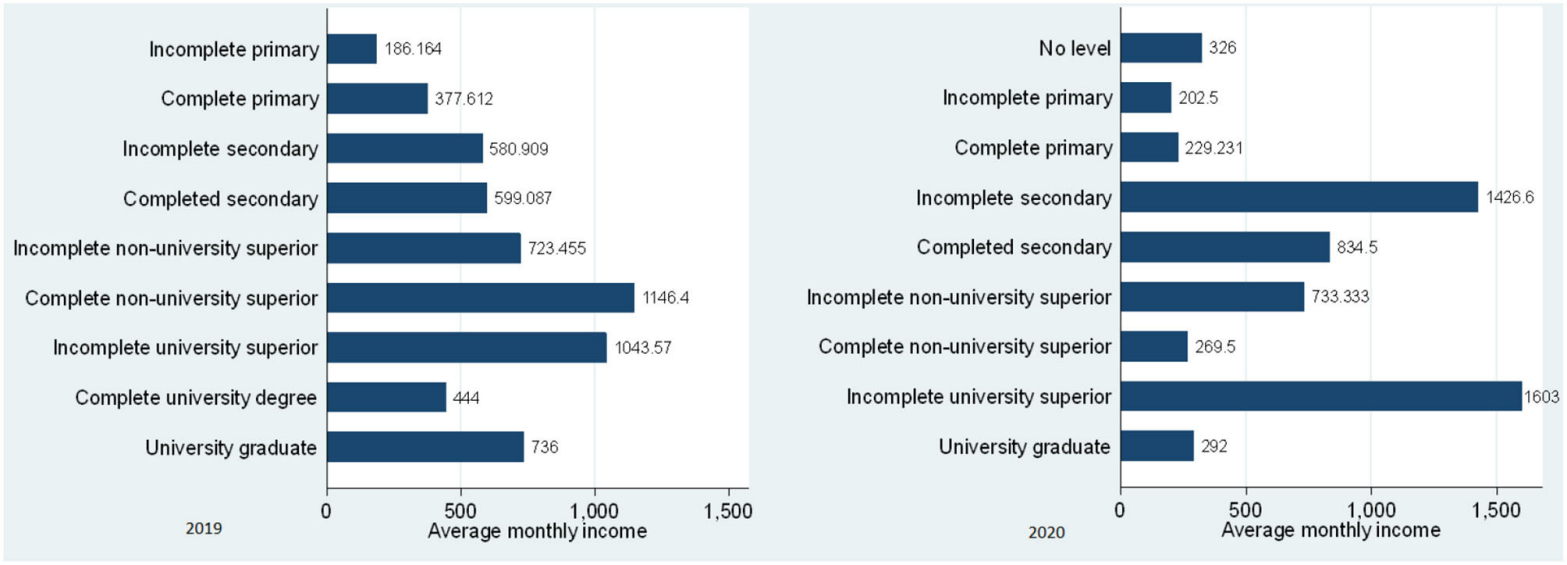
Average monthly economic income of self-employed workers by educational level, 2019–2020. Source: Own elaboration with information from ENAHO-INEI-2019 and 2020.

In this sense, the average income of independent workers by educational level for the scenario without pandemic (2019) and with pandemic (2020) is shown in Figure 3, where it is shown that there is a very significant income gap; this probably thanks to the productive differential that COVID-19 affected all the households, where most of the people with professional training entered the confinement phase, many of them losing the opportunity to function as independents, in view of the fact that social restrictions to avoid contagion greatly affected the normal development of activities. For example, a freelancer with a full primary school in 2019 managed to generate an average economic income of S/. 377.61 soles, while in 2020 that same worker managed to generate an average economic income of S/. 229.23 soles; in the case of the worker with a complete secondary education level in 2019, he generated an average economic income of S/. 599.08 soles and in 2020 increased their average economic income to S/. 834.50 soles (Figure 3).

In addition, the group most affected in this period of analysis was the group of independent workers with complete non-university higher professional training, since in 2019 they generated on average an economic income of S/. 1,146.40 soles and in 2020 it decreased to S/. 269.50 soles; in the case of the postgraduate professional training group, they were also affected, since in 2019 they generated on average an economic income of S/. 736.00 soles and in 2020 decreased to S/. 292.00 soles and this was one of the groups most affected by COVID-19, in view of the fact that the development of specialized training activities had a forceful stop due to the confinement, an issue that was gradually enabled through the development of remote activities (Figure 3).

### Inequality in the Economic Income of Independent Workers in the Puno Region in the Periods 2019 and 2020

To measure inequality in economic income, it is necessary to study the Gini coefficient; this indicator is between 0 and 1, where 0 corresponds to the perfect equality in economic income (everyone has the same income) and the value 1 explains a perfect inequality, where it shows that a group of people have all the economic income and the others none, thus showing a situation of maximum equality or distributive equity. Therefore, the Gini coefficient is based on the Lorenz curve, which is a graphical representation of an accumulated distribution function and is mathematically defined as the cumulative proportion of total income (y-axis), which obtains the accumulated proportions of the population (x-axis).

**Figure 4:**
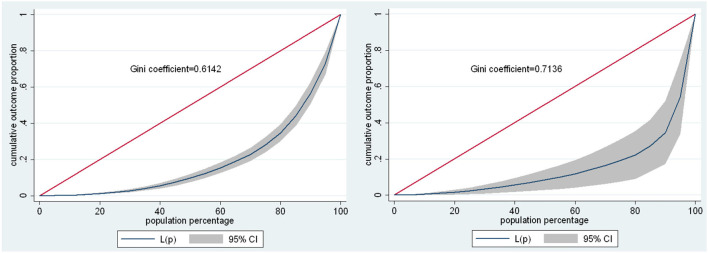
GINI coefficient of the self-employed—2019 and 2020. Source: Own elaboration with information from ENAHO-INEI-2019 and 2020. Where: G: Gini Coefficient; X: Cumulative proportion of the population variable; Y: Cumulative proportion of the income variable.

The Gini coefficient for the Puno region is consistent with the results obtained in the previous figures, given that in 2019 there was an inequality of economic income among independent workers (Gini = 0.6142) in relation to the national level (Gini = 0.415), since 0.61 is greater than 0.41. In 2020, inequality in economic income increased due to different aspects and mainly due to the limited opportunity that independent workers had due to COVID-19 problem, where the Gini coefficient amounted to 0.7136 in relation to the national level (Gini = 0.431); demonstrating in this way an increase in inequality in economic income and the gap in inequality that widened by 0.0994 (0.7136–0.6142), which shows that thanks to COVID-19 the economic income of independent workers in the Puno region has decreased and the most affected sector was agriculture, trade, and services, since it enters a stage of confinement and despite having a considerable level of educational training, the most affected were those with the lowest educational level and those who operate mainly in the marginal urban and rural areas of the districts and provinces (Figure 4).

### Social Determinants of Inequality in Economic Income in Self-Employed Workers in the Puno Region in the Periods 2019 and 2020

To identify the social determinants of inequality in economic income, logarithms were applied to the variable income of the independent worker and then the ordinary least squares model was applied, considering the traditional Mincer equation, considering the most representative variables in the families of these groups; therefore, the variables that explain the inequality in economic income are the age of head of household, the level of education achieved, and the gender of the independent worker (Table 2).

**Table 2:** Linear regression model for determinants of the economic income of the independent worker.

**Income of the self-employed**	**Regression coefficient, 2019**	**95% confidence probability**	**Regression coefficient, 2020**	**95% confidence probability**
Age of the self-employed	0.05193	0.0172	0.02066	0.007
Age^2^ of the self-employed worker	−0.00064	0.0086	−0.0001	0.008
Level of education of the self-employed	0.207418	0.001	0.348655	0.004
Sex of the self-employed worker	0.370082	0.009	0.141966	0.007
Constant	3.214908	0.002	2.932296	0.001
F-statistical	6.6		2.98	
Prob>F	0.0001		0.0298	
R-squared	0.1127		0.2255	
MSE Root	1.4549		1.2002	

According to Table 2, the statistics obtained for the years 2019 and 2020 are significant individually and together, since the T-calculated and the F-calculated are greater than 2 in absolute value with a significance level less than 5%, the R-square is 11.27 and 22.55% and the MSE root are equal to 1.49 and 1.20, which shows that both the models obtained for these periods are consistent.

In this sense, it can be established that the determinants of the economic income of the independent worker in the region of Puno are the age of the independent worker, level of education of the independent worker, and the sex of the independent worker. When analyzing the age of the independent worker, this directly influences the monthly economic income of this, in view of the fact that, at an increase of 1 year in the age of the independent worker, then the monthly economic income increases by 5.19% for 2019 and by 2.06% for 2020, evidencing a decrease in these periods by 3.13% (Table 2).

In the case of the level of education of the independent worker, this positively influences the monthly economic income of the independent worker; since, to an increase in the level of education of the worker, then the monthly economic income of these increases by 20.74% in 2019 and by 34.86% by 2020, showing an increase in economic income of 14.12% (Table 2).

With regard to the gender of the independent worker as head of the family, it was and has been very important, where despite the fact that in its largest proportion the independent workers are men and women played a decisive role in facing expenses at home in times of pandemic, which shows that the sex of the independent worker directly influences the economic income of the independent worker. Therefore, if the sex of the independent worker is male, then the monthly economic income increases by 37.00% in 2019 and by 14.19% in 2020, showing a decrease in monthly economic income of 22.89% between these periods; all this looking for other options for job creation, despite the fact that the labor market has still been very restricted and job opportunities are still very critical for that group of people independent that is mostly engaged in the activity of trade, agricultural and livestock activity, and services.

## Discussion

In this sense, according to the results obtained in this research, it is remarkable what is evidenced in the issue of the determinants of inequality in the economic income of the self-employed and this complemented with the results of the Gini coefficient and the gaps in income by levels of education and gender and all these results coincide with what is poured by Villar-mayuntupa and Villar-mayuntupa (2020), the same ones who considered that the effects of the pandemic at the level of Peru were aggravated by the existence of income inequality, in view of the fact that the Gini coefficient is close to 70%, and those who have been most affected are the population group that supports their economic income daily for the acquisition of goods and services; what is reinforced with what is determined by Jaramillo and Ñopo (2020), since the stagnation of the country's economic activities brought complications in the development of the generation of economic income of the group of the independent workers.

As determined by a study of Filgueira et al. (2020), we can also indicate that a large part of the employed population that was affected by social isolation due to COVID-19 showed a high risk of being left without income, being affected and showing significant inequality, contributing to the conditions to be able to face the necessary expenses; so, these depended on social characteristics such as age and level of education, a question that was also proven by this study.

By demonstrating that the determinants of economic income in the self-employed are sex, age, and level of education, it is consistent with what is determined by Witteveen (2020), since I also determine that the existence of gaps in economic income are mainly due to structural inequalities in sex, economic income by type of occupation, and complementarily considers the characteristics of women, highlighting the racial and ethnic aspects that they have.

In addition, we share with what is determined by Kim and Kim (2021), since for the group of independent workers and other groups, the income-health gradient was very strong in countries that had the highest number of deaths due to COVID-19 and this is what happened in Peru and in the Puno regions. In addition, it demonstrated the same as in this research, income inequality worldwide exacerbates the unequal health consequences of COVID-19 for older segments of the population, especially vulnerable to the disease.

## Conclusion

It was determined that there is a very significant income gap by educational level, thanks to the productive differential that COVID-19 affected all the households, where the independent worker with complete primary school in 2019 managed to generate an average economic income of S/. 377.61 soles, while in 2020 it managed to generate an average economic income of S/. 229.23 soles; in the case of the worker with a complete secondary education level in 2019 generated an average economic income of S/. 599.08 soles and in 2020 increase to S/. 834.50 soles; but in the group of independent workers with complete non-university higher professional training in 2019, these generated on average an economic income of S/. 1,146.40 soles and in 2020, which decreased to S/. 269.50 soles. In the case of independent workers with postgraduate professional training either with a master's degree/doctorate in 2019, they generated on average an economic income of S/. 736.00 soles and in 2020 decreased to S/. 292.00 soles.

There is inequality in the economic income of independent workers in the Puno region in the periods of analysis, since in 2019 there was a greater inequality of economic income among independent workers (Gini = 0.6142) compared to the national level (Gini = 0.415). On the contrary, in 2020, there was an increase in economic income inequality; this is due to different aspects and mainly due to the limited opportunity that independent workers had due to COVID-19 problem, where the Gini coefficient amounted to 0.7136 in relation to the national level whose Gini coefficient was 0.431; thus, demonstrating an increase in income inequality and the inequality gap that widened by 0.0994.

The determining factors of the economic income of the independent worker in the region of Puno are the age of the worker that explains in 5.19% for 2019 and in 1.72% for 2020, the level of education of the worker that explains in 20.74% for 2019 and in 34.86% for 2020, and the sex of the independent worker that explains in 37% for 2019 and in 14.19% for 2020, respectively.

## Data Availability Statement

The original contributions presented in the study are included in the article/supplementary material, further inquiries can be directed to the corresponding author/s.

## Author Contributions

JQ: conceived and carried out the study. GF and DC: study mentors. CY, WV, SA, BQ, NQ, and BC: participated in the design, performance of the data analysis, and in the drafting of the research work. All authors reviewed and approved the research work.

## Conflict of Interest

The authors declare that the research was conducted in the absence of any commercial or financial relationships that could be construed as a potential conflict of interest.

## Publisher's Note

All claims expressed in this article are solely those of the authors and do not necessarily represent those of their affiliated organizations, or those of the publisher, the editors and the reviewers. Any product that may be evaluated in this article, or claim that may be made by its manufacturer, is not guaranteed or endorsed by the publisher.
